# Oral Ondansetron versus Domperidone for Acute Gastroenteritis Associated Vomiting in Young Children

**DOI:** 10.7759/cureus.5639

**Published:** 2019-09-12

**Authors:** Hamza Hanif, Hassam Jaffry, Fatima Jamshed, FNU Amreek, Naresh Kumar, Wajid Hussain, Amber Rizwan

**Affiliations:** 1 Internal Medicine, Jinnah Sindh Medical University, Karachi, PAK; 2 Pediatrics, Jinnah Sindh Medical University, Karachi, PAK; 3 S. Arthur Localio Laboratory, New York University School of Medicine, New York, USA; 4 Medicine, Dow University of Health Sciences (DUHS), Karachi, PAK; 5 Pediatric Medicine, National Institute of Child Health, Karachi, PAK; 6 Family Medicine, Dr. Ruth Pfau Hospital, Karachi, PAK

**Keywords:** ondansetron, domperidone, acute gastroenteritis, pediatrics, young children, randomized controlled trial, open label trial, vomiting, antiemetic therapy

## Abstract

Introduction

The management of vomiting and antiemetic therapy in young children with acute gastroenteritis (AGE) has not been standardized by any management guidelines. Antiemetic drugs including promethazine, prochlorperazine, metoclopramide, ondansetron, and domperidone are readily used in the emergency departments (EDs). The aim of this study was to compare the efficacy of ondansetron with domperidone in cessation of vomiting in pediatric AGE.

Methods

This open-label, two-arm trial was conducted in a pediatric ED in Pakistan. Children of age 1 to 60 months presenting with acute vomiting and no or mild-to-moderate dehydration associated with AGE were randomized into two groups. Group A children received ondansetron suspension orally at a dose of 0.15 mg/kg body weight. Group B received domperidone suspension orally at a dose of 0.5 mg/kg body weight. The primary outcome was the number of children in each group who did not have any episode of vomiting 24 hours posttreatment. The data were entered and analyzed using Statistical Package for the Social Sciences (SPSS) for Windows version 20.0 (IBM Corp., Armonk, NY).

Results

At 6 hours, 87% of children in the ondansetron group improved and their vomiting episodes ceased as compared to 81% of children in the domperidone group. The differences were statistically insignificant (p>0.05). At 24 hours, 95% in the ondansetron group had improved and only 85% in the domperidone group. The results were statistically significant favoring the end results of the ondansetron (p=0.01).

Conclusions

This study concluded that ondansetron is more efficacious than domperidone in cessation of vomiting associated with AGE and no or mild-to-moderate dehydration in children of age three months to five years.

## Introduction

According to the United Nations Children’s Fund (UNICEF), although the rate of global under-five mortality has reduced to half in the last three decades, there is still great disparity between low-to-middle and high-income countries. Among preventable causes of under-five mortality, infectious diseases including diarrhea and pneumonia are the prominent ones [1]. Acute gastroenteritis (AGE) is a major cause of childhood morbidity and mortality in developing countries. In a global epidemiological report, there were 7.6 million global under-five mortalities; 64% of these were infective in cause. Pneumonia (14·1%), diarrhea (9·9%), and malaria (7·4%) were the leading infectious causes [2]. The frequency of AGE in under-five children has been reported from various low-to-middle income countries. The prevalence of AGE was 21.7% in India , 36% in Nepal, and 29-37% in Pakistan [3-5].

Vomiting is the most common and worrisome clinical presentation of AGE. It is distressing for the child as well as the caregiver and, if severe and intractable, may cause dehydration, may require emergency hospital admission, and can also be fatal. Oral rehydration therapy (ORT) is recommended in patients with mild-to-moderate dehydration caused by AGE [6]. Antiemetic therapy is not recommended for AGE by American Academy of Pediatrics and National Institute for Health and Clinical Excellence as one of its side effects include diarrhea [7-8]. However, these societies do encourage robust data to establish the efficacy of antiemetic therapy in acute diarrhea.

At present, no guidelines govern the management of vomiting and regulate antiemetic therapy in children with AGE. Antiemetic drugs including promethazine, prochlorperazine, and metoclopramide are used to manage vomiting in AGE. Domperidone - a dopamine antagonist - is also readily used despite its low efficacy. Ondansetron - a serotonin antagonist - is approved for the treatment of nausea and vomiting and has better side effects profile [9]. The end result with both domperidone and ondansetron has been comparable in randomized controlled trials (RCTs) [9-11].

The evidence of domperidone and ondansetron for the management of vomiting in pediatric AGE is still not concrete enough to draw conclusions. Furthermore, to date, there is no published literature regarding the usage of ondansetron or domperidone for the management of AGE in Pakistan. In light of this, we conducted an open-label prospective study to compare the efficacy of ondansetron with domperidone in cessation of vomiting in pediatric AGE.

## Materials and methods

An open-label, two-arm trial was conducted in the emergency department (ED) of a public tertiary pediatrics hospital in Pakistan from January to December 2018 after attaining approval from the institutional review board.

Non-probability consecutive sampling technique was adopted. All children under the age of 5 years with three or more non-bilious, non-bloody vomiting episodes within 24 hours, with signs suggestive of AGE such as diarrhea, abdominal pain, bloating or discomfort, with or without fever, were included. Consent was attained from the parents/guardians accompanying them.

Exclusion criteria included children who had already been given any antiemetic medication within 4 hours prior to enrolment, children with an underlying disease such as renal, liver diseases, congenital heart disease, neurological diseases, malignancy, immune deficiency, history of abdominal surgery, and diabetes, children severely dehydrated requiring intravenous (IV) fluid replacement, children severely malnourished, children with history of allergy to any antiemetic medication, children not tolerating oral feed, and children of parents/guardians who refused to participate.

All children under the age of 5 years, presenting with AGE at the ED were screened for enrolment in the study. Children fulfilling the above-mentioned inclusion and exclusion criteria were enrolled. A computer-generated table was used to randomize the enrolled children into two groups. Group A children received ondansetron suspension orally at a dose of 0.15 mg/kg body weight. Group B received domperidone suspension orally at a dose of 0.5 mg/kg body weight.

Both groups were given the study drugs within the ED. If a child had an immediate episode of vomiting following the administration of the medication, a second dose of the assigned antiemetic was given within 15 minutes. Thirty minutes after the administration of medication, children were allowed to take any fluid orally. Children were observed for 6 hours after taking the assigned medication. ORT was attempted when children were vomiting-free for at least 45 minutes. Those tolerating ORT in both groups were sent home with the assigned oral anti-emetic medicine. At the time of discharge from the ED, parents were advised to give the assigned antiemetic at the same dose if their children have nausea or ongoing vomiting but only in intervals greater than 8 hours. Further, they were advised to bring their children after 24 hours of treatment for reassessment.

For those who were still vomiting after 6 hours of initial treatment, hydration status was reassessed and decision on their hospital admission was made. Those who were severely dehydrated because of vomiting were admitted for management and excluded from the study. At the time of follow-up visit, parents were asked about the number of vomiting episodes during the last 24 hours to see if they had any episode of vomiting. If there was cessation of vomiting after 24 hours their antiemetics were discontinued and they were advised to continue ORT. For those still having vomiting, hydration status was reassessed and decision regarding their admission was made. The primary outcome was the number of children in each group who did not have any episode of vomiting 24 hours posttreatment.

The data were entered and analyzed using SPSS for Windows version 20.0 (IBM Corp., Armonk, NY). For continuous variables such as age, number of episodes of vomiting, mean and standard deviation (SD) was calculated. Frequencies and percentages were measured for categorical variables such as gender, primary outcome (cessation of vomiting episodes). To compare the frequency of primary outcome (cessation of vomiting episodes) between the two groups, ondansetron and domperidone, Chi-square test was used and p-values were calculated. P-value ≤0.05 was considered as significant.

## Results

Two hundred and eighty-two children were enrolled in the study. Twenty-seven children were excluded due to severe persistent dehydration and needed admission. Fifteen children were lost to follow up. Hence, the study was completed by 240 participants. Patients were randomly assigned into two groups - the ondansetron group included 123 (51.3%) children and the domperidone group included 117 (48.7%) children. Among these participants, there were 119 (49.5%) male and 121 (50.4%) female children. The mean age of these participants was 29 ± 16 months (range: 3-57 months). Their characteristics at the start of the trial are shown in table 1.

**Table 1:** Baseline characteristics of the study participants (N=240)

Baseline Characteristics	Ondansetron group (n=123; 51.2%)	Domperidone group (n=117; 48.7%)
Age, months	28 ± 15 (5-57)	27 ± 19 (3-53)
Male	66 (53.7%)	53 (45.3%)
Female	57 (46.3%)	64 (54.7%)
Body weight, kilograms	13.4 ± 5.7 (5.5-17.8)	13.9 ± 6.2 (4.8-16.2)
Height, centimeters	96.5 ± 21.4 (63.7-113.4)	95.3 ± 20.5 (59.4-107.8)
Duration of diarrheal episodes, hours	9.5 ± 4.8 (3.3-15.5)	8.1 ± 5.2 (3.0-18.5)
Number of diarrheal episodes (in the last 24 hours)	5 ± 3 (2-11)	7 ± 4 (2-12)
Duration of vomiting episodes, hours	6.8 ± 2.6 (2.5-8.3)	6.8 ± 2.6 (2.5-8.3)
Number of vomiting episodes (in the last 24 hours)	7 ± 4 (3-12)	6 ± 3 (2-14)
No dehydration	38 (30.9%)	45 (38.5%)
Mild to moderate dehydration	85 (69.1%)	72 (61.5%)
Second dose of antiemetic needed within 15 minutes	18 (14.6%)	26 (22.2%)

At 6 hours, 87% of children in the ondansetron group improved and their vomiting episodes ceased as compared to 81% of children in the domperidone group. The differences were statistically insignificant (p>0.05). At 24 hours, 95% in the ondansetron group had improved and only 85% in the domperidone group. The results were statistically significant favoring the end results of the ondansetron (p=0.01) (table 2).

**Table 2:** Comparison of vomiting outcome of ondansetron and domperidone group at 6 hour and 24-hour interval (N=240)

Treatment group	At 6 hours		P-value	At 24 hours		P-value
	Vomiting Cessation n (%)	Vomiting Persistence n (%)		Vomiting Cessation n (%)	Vomiting Persistence n (%)	
Ondansetron group	107 (86.9%)	16 (13.1%)	0.21	117 (95.1%)	6 (4.9%)	0.01
Domperidone group	95 (81.2%)	22 (18.8%)		100 (85.5%)	17 (14.5%)	

None of the children of the ondansetron group had a diarrheal episode during the ED stay, 11 (8.9%) had vomiting episodes within the first 6 hours, and none had any vomiting episode after 6 hours. In the domperidone group, two (1.7%) patients had one or more diarrheal episode and 18 (15.4%) patients had one or more vomiting episode during the first 6 hours of ED stay. Children receiving ondansetron spent a mean duration of 3.5 ± 2.8 hours (range: 2-7 hours) in the ED and children receiving domperidone spent 3.8 ± 3.6 hours (range: 3-8 hours). None of the differences were statistically significant (p>0.05).

When ORT was attempted in the patients, there were 12 (9.7%) children in the ondansetron group who did not tolerate it in the first go as compared to 23 (19.6%) in the domperidone group (p=0.02). Both groups did not report any adverse event. There was no episode of diarrhea exacerbation. Both drugs were safe and well-tolerated.

## Discussion

In this open-label comparative trial, ondansetron showed better efficacy in cessation of vomiting at 24 hours interval. At a shorter interval of 6 hours, ondansetron showed insignificant superiority. This study holds its value in being the first comparative trial from Pakistan. The study was conducted for an entire year, hence, it includes gastroenteritis cases with all seasonal variation. However, its major limitation is its open-label design. Concrete conclusions can only be established through longitudinal, randomized, double-blind trials. Furthermore, this study did not include a placebo group, hence, we cannot comment on vomiting cessation without any antiemetic intervention.

Previously, RCTs have shown a non-significantly higher proportion of cessation of vomiting with domperidone plus ORT as compared to ORT alone (79% vs. 73%) and also as compared to placebo or metoclopramide [10-11]. Even in this study, domperidone showed improvement in more than 80% of children even within the first 6 hours.

On the other hand, the trials which compared domperidone with ondansetron have deduced controversial results. At 24-hour follow-up, Rerkshuppaphol et al. reported cessation of vomiting in insignificantly higher percentage of children in the ondansetron group as compared to the domperidone group. Ondansetron group required significantly fewer drug doses during the 24-hour period as compared to the domperidone group for cessation of vomiting [9]. Significant reduction in the rate of hospital admission and the need for IV rehydration has also been reported with ondansetron [12]. In a multicenter double-blind RCT conducted by the Study ONdansetron vs DOmperidone (SONDO) Investigators group, a single dose of ondansetron was enough to successfully tolerate oral rehydration, reduced the duration of hospital stay to half, and also reduced the in-hospital episodes of vomiting as compared to domperidone [13]. These findings were also reinforced by the work of Freedman et al. where a single dose of oral ondansetron was very effective in reducing vomiting episodes and facilitating oral rehydration [14].

Conversely, in a two-hospital, double-blind, placebo-controlled, ED-based, RCT conducted in Pakistan, patients were randomly assigned to single-dose oral ondansetron or placebo. The primary endpoint was IV rehydration within 72 hours of randomization. IV rehydration use was 12.1% children in the ondansetron group and 11.9% in the placebo (odds ratio [OR] 0.98). Bolus fluid administration was required within 72 hours of randomization in 10.8% in the placebo group and 10.3% in the ondansetron group (OR 0.95). Hence, oral administration of a single dose of ondansetron failed to improve the clinical outcome and reduce the rate of IV rehydration administration in non-dehydrated children with diarrhea associated with vomiting [15]. Their methodology was robust as it was double-blind and included a larger sample size. However, as compared to our study, their work was placebo-controlled and their results are only valid for children with no dehydration. We recommend our results to be generalized for children no and mild-to-moderate dehydration due to diarrhea and vomiting. In another local placebo-controlled trial, children with mild-to-moderate dehydration with diarrhea and vomiting with administered oral ondansetron, the treatment group showed earlier and better rehydration and a significant reduction in vomiting episodes [16]. Other regional trials have also shown significantly better clinical outcome with ondansetron as compared to domperidone in acute vomiting [17-18].

The role of oral ondansetron in cessation of vomiting in young children is still controversial. As suggested by Golshekan et al., we also recommend robust, placebo-controlled trials to establish a standard protocol for the management of vomiting associated with AGE in young children [18].

## Conclusions

This study concluded that ondansetron is more efficacious than domperidone is cessation of vomiting associated with AGE and no or mild-to-moderate dehydration in children of age three months to five years. Oral ondansetron is a useful antiemetic for emergency management of vomiting, however, the need for robust clinical trials to establish standard protocols still persists.
